# Conception and Interpretation of Interdisciplinarity in Research Practice: Findings from Group Discussions in the Emerging Field of Digital Transformation

**DOI:** 10.1007/s11024-023-09489-w

**Published:** 2023-02-25

**Authors:** Josephine B. Schmitt, Anne Goldmann, Samuel T. Simon, Christoph Bieber

**Affiliations:** 1 Center for Advanced Internet Studies, Bochum, Germany; 2 University Duisburg-Essen, Duisburg, Germany; 3 Ruhr University Bochum, Bochum, Germany

**Keywords:** Interdisciplinarity, Collaborative knowledge production, Group discussions, Digital transformation research

## Abstract

In recent years, we have been observing the phenomenon of an emerging scientific field: *digital transformation research* (DTR). Due to the diversity and complexity of its object of research digital, transformation is not effectively researchable if confined to the boundaries of individual disciplines. In the light of Scientific/Intellectual Movement theory (Frickel and Gross 2005), we wonder how interdisciplinarity could and should be mobilized to further advance the development of the field of DTR. To answer this question, we (a) need to understand how interdisciplinarity is conceived and (b) how it is considered in research practice by researchers in the emerging field. This is important, as scientists’ application of interdisciplinarity will highly influence an emerging field, shape its growth, consolidation as well as its academic establishment. We conducted six group discussions with 26 researchers from different disciplines and career levels (PhD students, postdocs, professors). The discussions were studied with a structuring qualitative content analysis. The results reflect the vagueness of the concept of interdisciplinarity. Interdisciplinarity is largely conceived as multidisciplinarity. Further, the interviewees mentioned more challenges than opportunities when it comes to interdisciplinary DTR. The present study widens the scientific understanding about how researchers of different career levels perceive, learn, and practice interdisciplinarity in DTR. It further provides valuable indications of how interdisciplinary research in an emerging field can be profitably shaped for practice.

The dynamics of digital transformation with disruptive technological upheavals and serious social consequences permeates all areas of life. Consequently, digital transformation as a ubiquitous, complex phenomenon is not effectively researchable if confined to the narrow boundaries of individual disciplines. In recent years, we have been observing the phenomenon of an emerging scientific field: *digital transformation research*^1^ (DTR). DTR is very dynamic and increasingly complex. It includes research of implications and effects of social, political, economic, cultural, and technical changes that accompany digitization. Due to the diversity and complexity of its object of research, DTR necessarily interlinks different disciplinary perspectives from the social sciences and humanities to computer and technical sciences. However, until now, at least in Germany, this field is mainly shaped by the social sciences.

The formation of a new scientific field is a historically well-known phenomenon in higher education. It can be understood as a social and intellectual movement (SIM; Frickel and Gross 2005; Jacobs and Frickel 2009) triggered by critical experiences or events (e.g., technological innovations, societal disruptions). As SIM, field development is dependent on local social, political, and cultural context, resulting in different variations of legitimization, institutionalization, and resource allocation (e.g., money, attention). Many modern disciplines started as interdisciplinary movements (Jacobs and Frickel 2009; Stock 1989) using the advantage of being able to easily switch between different bodies of knowledge, theories, data, and methods, negotiating disciplinary boundaries and setting new epistemic standards. The ubiquity and complexity of digital transformation in itself is the basis of the border-crossing nature of DTR. The historical evolution and therefore conceptual malleability of computational tools for science is further an important reason for a multitude of disciplinary viewpoints in DTR.

Against this background, we wonder how interdisciplinarity could and should be mobilized to further advance the development of the field of DTR—of course, keeping potential challenges of interdisciplinary collaboration in mind (e.g., Leahey et al. 2017). To answer this question, we (a) need to understand how interdisciplinarity is conceived and (b) considered in research practice by researchers in the emerging field. This is important, as scientists’ conceptualization and application of interdisciplinarity will highly influence an emerging field, shape its movement, growth, consolidation as well as its academic establishment. We address these issues through a qualitative analysis of group discussions with researchers on digital transformation.

Previous research suggests that in dealing with interdisciplinarity in field development it is important to also pay attention to the researchers’ career level: While younger researchers are less invested in old paradigms and tend to be more open towards new interpretation of data, more experienced researchers have more social capital, perceive fewer professional risks and, thereby, more freedom to advance and stabilize the SIM (Frickel and Gross 2005). Further, research shows that scholars are differently affected by tensions between their willingness to engage in interdisciplinary research, disciplinary requirements, and the academic evaluation system depending on their career level (e.g., Milman et al. 2015; Rhoten and Parker 2004). Understanding expectations and experiences of researchers in different academic stages is an essential foundation for designing structures and measures to facilitate collaboration across disciplinary borders (Milman et al. 2015)—and, thereby, advancing an emerging field such as DTR.

We will examine the scientific discourse on interdisciplinarity and what is currently known about interdisciplinary research in practice before presenting and discussing the results of our study.

## Defining Interdisciplinarity

Defining interdisciplinarity can be a challenging endeavor as there are various asymmetries in the conceptual and theoretical perspective on interdisciplinarity, which cannot be dealt with here in all detail and depth. Interdisciplinarity can be understood as crossing disciplinary borders by mutual consent (Fischer 2011) *between* researchers of different disciplinary perspectives or *within* a researcher (interdisciplinary knowledge). Interdisciplinary research includes activities "which juxtapose, apply, combine, synthesize, integrate or transcend parts of two or more disciplines" (Miller 1982: 6) dealing with complex problems which require multiple forms of knowledge (Porter et al. 2006). This shifts the discussion away from an either-or-situation and toward a question of *how* to integrate disciplinary perspectives (Jasanoff 2004).^2^

The *how* is reflected in the thoughts on alternative approaches in knowledge production through expanding contexts, places, and evaluation criteria of academic research (Nowotny, Scott, and Gibbons 2001). This results in various perspectives on the *extent* of crossing disciplinary boundaries and integrating knowledge in interdisciplinary research. Fischer (2011), for example, distinguishes between *weak* and *strong* interdisciplinarity. While weak interdisciplinarity refers to the combination of different perspectives within one discipline (e.g., personality psychology and organizational psychology), strong interdisciplinarity means the combination of perspectives from different disciplines. With a stronger focus on practical applicability, Huutoniemi et al. (2010) differentiate between three modes of interdisciplinary research: scope, type, and goals. *Scope* refers to the conceptual and cultural distance (narrow vs. broad) between disciplines that collaborate to achieve a certain research *goal* (epistemological, instrumental, or mixed). The *type of interaction* relates to the extent of integration of disciplinary knowledge. Several individual researchers from various backgrounds cooperating under the framework of a common project, exchanging and pooling data, and ideas not in a dialogic but in a juxtaposing manner, for example, is understood as broad type of interdisciplinary interaction. Stember (1991) or Darbellay (2016), by contrast, regard this kind of interaction as *multi*disciplinarity—an underdeveloped antecedent of interdisciplinarity characterized by a lower degree of openness, interaction, and integration. These different perspectives on interdisciplinarity may have important consequences for how interdisciplinarity is used and practiced by researchers.

### Interdisciplinarity as Answer to Complex Problems

According to Defila and Di Giulio (1998), interdisciplinarity is "the answer to the 'dynamics of social problems’" (p. 131), which distinguishes it as a particularly suitable mode of knowledge production for dealing with complex and dynamic digital transformation issues. As such, interdisciplinarity is embraced by many scholars as driver of epistemic change and treated as a trend by policymakers, research institutions and funding bodies. The scientific discussion, however, swings between a wide range of definitions (as briefly shown above) and fierce criticisms. The discourse has come a long way from being declared a “spontaneous ideology […] oscillating between a vague spiritualism and technocratic positivism” (Althusser 1967: 97) and being juxtaposed to disciplinarity and trivialized through the dichotomy of “royal science” versus “nomadic science” (Deleuze and Guattari 1987: 367). Others argue for interdisciplinarity as a tool to counter challenges of disciplinarity. Suchman (2013), for example, points to the fact that an overly strong focus on a discipline may shift the attention to hierarchy and identity rather than focusing on content-related work. This builds on the criticisms that disciplinary structures may serve as an instrument for exercising power (Shumway and Messer-Davidow 1991), and thereby limiting innovation and progress of science, as well as reducing relevance for society (Barry and Born 2013; Crow 2010).

Even if there is some general confusion about the understanding and importance of interdisciplinarity, the number of institutions, programs, and administrative policies that embrace the ideal of knowledge co-production in interdisciplinary collaboration is notable (e.g., Wissenschaftsrat 2020). In the case of DTR this aspect is mirrored in the fact that in Germany, for example, *interdisciplinary* field development in DTR is strongly driven by political and academic organizations promoting scientific research. Although the field of DTR is still evolving, we already can observe various attempts to stabilize the field within specialized non-university organizations such as the Joseph Weizenbaum Institute (JWI), or the Center for Advanced Internet Studies (CAIS). These consider the interdisciplinarity of research on digital transformation as self-evident. According to Jacobs and Frickel (2009), there is mixed support for the notion that interdisciplinarity is something that can be manufactured from top-down by administrative policies. In the context of a SIM an interdisciplinary orientation of an emerging field rather can be characterized “as intellectual insurgencies generated and sustained from below by faculty and graduate students organizing collective challenges to the disciplinary order” (p. 57). Considering the importance of individuals in initiating and shaping such movements, it seems crucial to understand how they see interdisciplinarity, how they build connections and collaborate across disciplinary boundaries to further advance the development of the field.

### Interdisciplinarity in Research Practice

Currently, joint research projects on digital transformation bear weak traces of interdisciplinarity, better described as situations of loose cooperation without transcending borders between disciplines. At best, computer scientists are organized to a relation of subordination or service to social scientists when it comes to research questions that require a deeper technical understanding or simply programming skills (subordinate-service mode of interdisciplinarity, Barry et al. 2008). One reason for this can be an ambiguous understanding of interdisciplinarity. Cooke et al. (2020) have shown that among researchers engaging in interdisciplinary research, perspectives on interdisciplinarity are diverse. They range from perceiving interdisciplinarity as “meaningless academic buzzword” (ibid.: 143) to interdisciplinarity being an enrichment to ways of knowing, academic relationships, and new ways of problem solving. These ambiguities can have severe consequences for the quality and advancement of interdisciplinary research—and eventually hinder the developments of new fields (Huutoniemi and Rafols 2017; Madsen 2018). Lindvig and Hillersdal (2018) argue “that the lack of clarity in defining and evaluating interdisciplinarity became a way of organising research that produced a dominant, but vague, configuration of interdisciplinarity” (p. 42) reproducing existing monodisciplinary research and power structures. Against this background, we ask:

#### Research Question 1

How do researchers in the emerging field of DTR understand interdisciplinarity?

Interdisciplinary collaboration is attributed with carrying innovative potential and being necessary to tackle complex fields. As the additional demand for being interdisciplinarily skilled is challenging for some researchers, it results in the sub-par adoption of a rather multidisciplinary than interdisciplinary organization of research (Rhoten 2003). Researchers are confronted with various tensions between the willingness to engage in problem-oriented interdisciplinary research, disciplinary constraints, and the academic evaluation system (Rhoten and Parker 2004). Moreover, research activities in an emerging field are embedded in an atmosphere of striving for legitimacy and acceptance inside and outside of a potential scientific community (Debackere and Rappa 1994) adding additional uncertainties.

Apart from that, interdisciplinarity is not only a mode of research and knowledge creation but also a social practice (Castán Broto, Gislason, and Ehlers 2009). As such, it comes with various communication difficulties (Bridle et al. 2013) which require cultural and social competencies in researchers to value diversity in research teams (Reich and Reich 2006). The integrative capacity of a science team may facilitate social and cognitive integration processes necessary for effective team-work and innovative outcomes (Salazar et al. 2012). These aspects underscore the need to create frameworks in which benefits of interdisciplinary research can unfold and tensions are kept low—or researchers are enabled to deal with the latter. Against this background, a perceptual approach seems crucial to understand perceptions and experiences of digital transformation researchers to further advance the field. Thus, we ask the following:

#### Research Question 2

How do digital transformation researchers conceive and consider challenges and opportunities of interdisciplinarity in their research practice?

There may be individual differences between researchers of how opportunities and challenges of interdisciplinary collaboration are regarded—and later tackled in research practice. Studies have shown that collaborative research leads to papers of high quality and scientific impact (e.g., Bu et al. 2018; He et al. 2009)—especially for researchers of lower career levels. However, the scientific impact of interdisciplinary papers depends on how they are evaluated within the participating disciplines (e.g., Rafols et al. 2012). Consequently, especially early-career researchers see interdisciplinarity as barrier to individual career planning (Rhoten and Parker 2004), a fact that may be problematic for the successful formation and advancement of a new interdisciplinary field. This is in line with considerations by Frickel and Gross (2005), who credit more experienced researchers with more freedom and power to advance and stabilize a growing field as they face fewer professional risks—yet they need cooperation with younger scientists as those are more avant-gardist, when it comes to new forms of methodological innovations. Thus, the effort of “stabilizing the field” (Frickel and Gross 2005) is a cooperative endeavor, combining the efforts of researchers on all stages of academic careers. This rationale leads us to answer both research questions with special regard to different career stages.

## Method

Six group discussions with researchers focusing on research on digital transformation of about 90 minutes were conducted. Group discussions are particularly suitable for exploring complex topics about whose structure little is known (Kühn and Koschel 2018). Due to the COVID-19 pandemic discussions were conducted online via the video-conferencing tool *Zoom* following the recommendations for online focus groups by Forrestal and colleagues (2015).

### Sample

Discussion groups (ad hoc groups) were homogeneous in terms of the participants’ academic career level (PhD student, postdoc^3^, professor). As recommended by Kühn and Koschel (2018), two group discussions per target group were conducted. Relevant persons with different disciplinary perspectives on digital transformation at different German research institutions (e.g., universities, non-university research institutes) were directly approached. Discussions were conducted between 14 September and 01 October 2020 (*n*_female_ = 13, *n*_male_ = 13). Table 1 provides an overview of the disciplinary composition of the groups.

**Table 1:** Overview about the participants’ disciplines by group

		Interviewee	Discipline
Professors	G1	B1	Media Studies
		B2	Computer Science
		B3	Economics
		B4	Psychology/Computer Science
	G2	B5	Communication Studies/(Media-)Psychology
		B6	Linguistics
		B7	Philosophy
Postdocs	G3	B8	Political Science
		B9	Political Science
		B10	Computer Science
		B11	Political Science
	G5	B17	Political Science
		B18	Media Studies
		B19	Business Information Systems
		B20	Communication Studies/(Media-)Psychology
		B21	Media and Communication Studies
PhD Students	G4	B12	Psychology
		B13	Philosophy
		B14	Communication Studies
		B15	Political Science
		B16	Legal Studies
	G6	B22	Political Science
		B23	Communication Studies
		B24	Communication Studies
		B25	Business Information Systems
		B26	Computer Science

B16. Legal Studies: Due to technical difficulties, the person could only participate for a very limited period.

### Procedure

Discussions were each led by two trained researchers—one male, one female. Participants were informed in advance about the recording of the discussions, verbal consent was obtained from all. Adding to the oral exchange, text-based methods were used as recommended by Baudendistel et al. (2015). Prior to the discussions, participants received a link to a shared online whiteboard (via the collaborative note-taking tool *Mural*). They were asked to note initial thoughts on opportunities and challenges of interdisciplinary research. During the group discussions, these notes could then be collaboratively changed and extended. Each discussion was characterized by the alternation between individual work, group interview phases, and discussion rounds. The interview guidelines in the online appendix^5^ provide a detailed overview of the process. Discussions were recorded and the soundtrack transcribed by a professional service provider (for transcription rules, see Clausen, Jankowski & Dawid 2020).

### Analysis

The interviews were analyzed by means of a structuring qualitative content analysis proposed by Kuckartz (2018) using MAXQDA 2020 (VERBI GmbH). Structuring qualitative content analysis was particularly suitable as the focus was on identifying topics and sub-topics in the context of interdisciplinarity, as well as their systematization and possible mutual relations. The minimum coding unit was one sentence. Based on the interview guidelines and in correspondence with the research questions, only a few main categories were formed a-priori. During an initial coding of the entire material, further categories and subcategories were determined inductively. Concept-driven categories as main categories were combined with data-driven categories as subcategories (Schreier 2014). This process was repeated several times until a differentiated system of categories was formed which is structured through thematic categories. As recommended by Elo and colleagues (2014), one researcher was responsible for the analysis and one other carefully followed up on the categorization and coding process. Diverging opinions were continuously discussed. The code book is added in the online appendix^6^.

## Results and Discussion

### Understanding Interdisciplinarity

Regarding RQ1, three superordinate categories are crucial: conceptional understanding, motivation for interdisciplinarity, learning interdisciplinarity. In the postdoc group all three categories are quite evenly distributed. Both PhD students and professors have more strongly addressed the topic of motivation than learning and conceptual understanding^7^.

#### Interdisciplinarity: Ambiguous Concept or Lack of Understanding?

Participants highlighted the conceptual vagueness of interdisciplinarity, which is also reflected in their research practice. On the one hand, this was explicitly expressed (PhD student, G4^8^): *"Where does interdisciplinarity actually begin? Does it start when I talk to a sociologist in the political sciences […] Or, at what distance are we interdisciplinary?"* On the other hand, conceptual vagueness was revealed implicitly as the term ‘interdisciplinary’ was used for describing very different contexts and research projects the researchers have been engaged in. These findings suggest that even those researchers who are engaged in—what they quite naturally might consider as—‘interdisciplinary research’ do not necessarily have a profound conceptual understanding. Alternatively, this highlights the conceptual ambiguity of interdisciplinarity found in the scientific discourse. Both can have severe consequences for the quality and advancement of interdisciplinary research (Huutoniemi and Rafols 2017; Madsen 2018)—especially in the context of an emerging scientific field depending on interdisciplinarity (Stock 1989).

Concerning the application of interdisciplinarity in day-to-day research, discussants had a rather coexistent than an integrative understanding—the former is mirrored in the concept of *multi*disciplinarity (Stember 1991) or at best as a very broad understanding of interdisciplinary interaction (Huutoniemi et al. 2010). Meaning, interdisciplinarity is less about overcoming disciplinary boundaries and integrating perspectives, knowledge and methods of other disciplines but about letting different disciplinary perspectives work side by side. According to Stember (1991), this hints at the interviewees’ low openness and low readiness for interaction. Increased openness, however, would be a prerequisite for successful scientific field creation (Woiwode and Froese 2020). The following quote (Professor, G1) represents this aspect fittingly: *"But I think it is also interdisciplinary when a person or a disciplinary homogeneous team tries to import theories or concepts or methods from other disciplines, to adapt them."* Taking the study’s sample into account, which mainly consists of social scientists, this finding is surprising, as Milman et al. (2015) have shown that social scientists tend to agree with an understanding of interdisciplinarity as an integrative co-creative mode of knowledge production.

An integrative—and according to Stember (1991)—more advanced understanding of interdisciplinarity is shown in the following quote by a PhD student (G4): *"The important point, however, with interdisciplinary collaboration is that all parties want to get something out of it and that in the end the sum of all parts is quasi better than the simple sum."* While the coexistent understanding of interdisciplinarity in DTR tends to predominate in all career stages, the differences seem to be smallest in the group of PhD students. Professors seldomly provided an integrative understanding (see Table 2 for a detailed overview). Arguably, today’s younger researchers have more opportunities for learning and experimenting with integrative approaches to interdisciplinarity, for example through the increasing number of interdisciplinary elements in teaching and research. Whereas individuals at higher career stages have been socialized more strongly into disciplinary contexts. Or, like Frickel and Gross (2005) put it regarding the development of new scientific and intellectual fields: “Generational swifts may occur in the personnel who comprise an intellectual field, with cadres of younger researchers less invested in old paradigms and more open to new interpretations of data […]” (p. 210). As DTR moves towards stability by establishing new, specialized research centers, those structures should ensure dialogue and cooperation across generations. Material from the group discussions suggests that especially an exchange between experienced and young researchers could open up innovative spaces for fertile cooperation. However, this also underlines the field’s capacity to advance in the direction of an integrative understanding of interdisciplinarity in research on digital transformation.

The prevalence of a coexistent understanding of interdisciplinarity in our sample coincide also to a significant extent with findings of Woiwode and Froese (2020). A genuine integration of knowledge and breaking down disciplinary boundaries is rarely achieved. Knowledge from other disciplines is more likely to be used to develop one’s own disciplinary structures or to expand one’s own career path. In the context of our group discussions, however, it is precisely this motivation to overcome boundaries that is seen as a prerequisite for advancing interdisciplinary DTR.

#### Motivation for Interdisciplinarity

Discussions indicate that topic-related motives as well as personal predispositions and structural incentives promote interdisciplinarity—partly contradicting previous findings that engaging in interdisciplinary research often is motivated by external factors such as work context, project funding, and publication opportunities (Carayol and Nguyen Thi 2005; Milman et al. 2015; Angelstam et al. 2013). Overall, professors show the most balanced naming of the different motivations to collaborate across disciplines (see Table 2 in the appendix). Compared to other career levels, they more frequently mention structure-related motivation as a reason for interdisciplinary DTR. For them, calls for funding significantly foster planning and carrying out interdisciplinary research projects (Professor, G2): *"Well, I also have to say quite profanely, often the motivation comes from the outside, for example, funding institutions […] the example of digital religion, where I’m working. I don’t think I would have gotten involved with this topic if there hadn’t been funds that were made available for such a large project."* However, such an incentivization structure also leads to greater demands on resources: *"In the end, it’s also an incentivizing structure […] where you really try to think outside the box. That takes more time and money than if I sit down alone for my discipline […]."* (Professor, G2)

Yet, one also must be *“the type”* for this (e.g., PhD student, G6; Professor, G1). Among the PhD students, topic-related and personal motivation are named quite evenly as relevant drivers of interdisciplinary collaboration. According to them, interdisciplinary DTR can only succeed if scientists are willing to transcend their disciplinary boundaries. The following quote supports this perspective (PhD student, G6): *"I believe that interdisciplinary work […] can only work if it is self-motivated and if it comes from the scientists themselves."*

#### Learning Interdisciplinarity

Personal initiative, organizational environment and conditions while studying are named as important for learning interdisciplinarity. Among the interviewed professors, all three are mentioned equally. Regarding personal initiative as a necessary condition, individual effort and engagement seems to be decisive (Professor, G2): *"Yes, you can certainly learn it! It usually involves a lot of work when you must learn the basic concepts of other disciplines. […] If you’re interested in issues that make it obvious that you can learn something from other disciplines, you’ll be willing to look into it."*

The lower the career level, the more often the time to obtain a university degree is mentioned as an important context for learning interdisciplinarity. Participants further see conducive organizational conditions within universities and research institutes as necessary. Such conditions, however, are not identified as sufficient (PhD student, G4): *"I believe that the focus of training or studies is now moving strongly in the direction of interdisciplinarity. However, […] it is something completely different to do interdisciplinary research than to have had an interdisciplinary course of studies."*

### Interdisciplinarity in DTR

Regarding the disciplinary composition of research teams in DTR, respondents identified a special need for computer scientists—a shortage which is also reflected in the study’s sample. In addition, social sciences, law, and psychology are mentioned as central disciplines to be included. Another aspect concerning the composition of the group is brought into play particularly by the professors. To their mind the participation of actors from outside of academia (e.g., citizens, economy, pedagogical staff) is necessary to ensure societal relevance of the emerging field. This is referring to a transdisciplinary approach which requires even more openness, interaction, and integration between the participating stakeholders (e.g., Stember 1991; Wickson et al. 2006).

However, according to the discussants, not only the composition of the research team is crucial but also how the different disciplines are appreciated in research activities. In DTR, “*[c]omputer scientists are seen as the auxiliary scientists. Of course, they don’t like that”* as one professor (G1) underlined. In a worst-case, different disciplines are reduced on single competencies which do not mirror their scientific potential as a professor exaggeratedly formulated (G1): *“We bring in the computer scientist and he programs us something nice. Or the other way around, we bring in the sociologists and they’ll reflect on the results.”*

In the following, we expand on results regarding RQ2 addressing the challenges and opportunities of interdisciplinary collaboration in DTR in more detail. We understand *challenges* to be the participants’ descriptions of what has caused interdisciplinary collaboration to fail and what is perceived as problematic in current research contexts. We include explanations of where obstacles to interdisciplinarity are expected. *Opportunities* are understood as descriptions of positive experiences and statements about where potential for interdisciplinarity in DTR is seen. We distinguish in both cases between individual and structural aspects^9^.

#### Challenges

Individual challenges include specific requirements that arise from the researchers’ interdisciplinary research context. Interdisciplinary collaboration must be learned in the first place. This requires a deeper understanding of one’s own discipline (Professor, G2): *“Above all, I think, the transition to interdisciplinary working [is challenging]. Because this whole knowledge of the strengths of one’s own discipline, explaining the concepts, that’s something you have to work on first."* A disciplinary understanding, in turn, is necessary for comprehending each other in terms of theories, data, and methods (Osborne 2013) as the following statement underlines (PhD student, G4): *“I've experienced this first-hand. In a project, I'm working together on a tandem PhD project with colleagues from IT-security. The problem was in the beginning that we didn’t speak the same language."* To address this problem in practice discussants suggest different measures, for example, a *“reading circle”* (Professors, G2) where papers from different disciplines were read and discussed to approach other disciplinary perspectives, or that in an interdisciplinary team *“each subject first presents itself, what it is good at. What it would like to learn from the other subject. And what typical prejudices are. That helps a lot. Also, to reduce uncertainties. Because what we very often have is that colleagues with a social science background think: 'I'm too stupid to understand the formula.' And the computer science colleagues think: 'I'm too stupid to understand all the technical terms.' To pick up on this uncertainty and show that in both cases it’s usually because the author simply hasn’t explained what he or she was thinking, and not at all because you somehow fundamentally can’t understand it. That’s totally helped.”* (Professors, G2)

The analogy of languages has already been discussed by Bauer (1990). He even argues that differences among researchers of different disciplines can be understood as *cultural* ones. Although everybody is engaged in finding the ‘truth’, they do not only differ in their perception of what ‘truth’ exactly means but also in attitudes, habits, and manners as well as the expression of it: *their language*. Acknowledging these differences by valuing that they might serve separate purposes is the “first requirement for useful interdisciplinary effort” (Bauer 1990: 113). As with learning a new language, also interdisciplinary collaboration could benefit from translation and interpretation. Consequently, interviewed researchers in our study wish for an external entity to support and foster this mutual understanding within an interdisciplinary research team. This added ‘*facilitator*’—a concept borrowed from business innovation—could bridge the communicative and processual gaps between fragmented disciplinary approaches—and hierarchies, which are also considered as limiting factor in interdisciplinary DTR (G2). Thereby, successful interdisciplinarity in DTR would not only depend on it being strongly situated within each individual but is being supported by an additional role ensuring structured support.

The importance to include further innovative structuring methods is mentioned by various participants. One postdoc underlines (G3): *“I think it’s clear that we’re trying to incorporate the principles of agile working and new work.”* A professor (G1) likewise suggests working with *“agile methods”* in DTR and emphasizes the challenge to apply them: *“In software development, agile methods are being used. Something like Scrum is probably familiar to everyone, at least in terms of the concept. And applying that to a research process or to apply it to a project where different disciplines work in this way is a great challenge.”* The idea of integrating concepts of business innovation in interdisciplinary research and how it fits to the above-formulated need of integrating a facilitator role will be expanded upon in the final discussion.

Also challenges of interdisciplinary research regarding career paths, respectively one’s own positioning in the academic landscape are expressed: *"So yes, asserting oneself, establishing oneself. But these are rather career-strategic questions. Or just the problem of how I find my niche and how I make a name for myself without somehow appearing to have a washed-out research profile."* (Postdoc, G3) For effective and solution oriented interdisciplinary DTR, researchers see the softening of the boundaries of traditional disciplines as necessary condition. This includes that career paths outside of established professional societies and traditional publication organs should be possible, or that there must be more openness on their part to interdisciplinary approaches (Müller and Kaltenbrunner 2019).

Structural challenges include disciplinary environments, their rootedness in the German science system, and their lacking openness. A further central structural challenge is the publication landscape. Although numerous interdisciplinary teams and research contexts are emerging, suitable outlets for DTR are often lacking, as one professor (G2) emphasized: *“I would really wish, especially for my collaborators, that there were more opportunities to publish interdisciplinary. I think that’s quite a task for journal designers*.” Following the ideas of SIM (Frickel and Gross 2005), the development of new publication outlets might reduce uncertainty on the researchers´ side and supports stabilizing an emerging field. Considering that interdisciplinary publications receive higher impact than disciplinary publications (van Noorden 2015) this is even more important—although disciplinary openness towards new outlets is necessary as well (Rafols et al. 2012). However, the lack of suitable outlets often results in disciplinary workarounds such as described in the following (Professors, G2): *“We have the one-paper-per-team deal. That is, when we have written a paper, which is very central to communication science, then the counter-deal, so to speak, would be that we try to export our expertise to computer science next.”*

Only a few respondents mentioned funding as a structural challenge. Although it is known that there are several grants for interdisciplinary research, it appears to be difficult to identify them or to set up projects that will ultimately be funded (e.g., Professor G1): *“I worked in different projects in the social and cultural sciences. And somehow together with computer scientists. […] That’s totally difficult to set up anything at all that’s funded, even if there are funding opportunities for it."*

#### Opportunities

Individual opportunities include statements emphasizing positive effects of interdisciplinary collaboration for researchers themselves. This perspective connects with the individual challenges outlined above—especially achieving a shared understanding. Once this understanding on theories, data and methods is established, interdisciplinary research can be enriching (Postdoc, G3): *"So if we only see it in terms of content: you can learn very, very well and very, very much from each other. This mutual inspiration I find basically the most exciting thing about it."* This shared understanding is best achieved by fostering in-depth exchange and conversation to overcome *“a bit of crunching”* between researchers of different disciplinary backgrounds as well as strengthening the relationships between them in regular in-person meetings (see e.g., Postdoc, G3).

Structural opportunities point out the positive aspects of interdisciplinarity for science, disciplines, and research projects including competitive advantages. Our participants emphasize that interdisciplinary DTR benefits from the strengths of the individual disciplines involved (PhD student, G6): *"Each discipline contributes what it does best. And I think that’s the central advantage of interdisciplinary work."* This combination of co-produced contributions can improve the quality of the research output. However, this is also depending on the actual relationship between the disciplines. As outlined above, DTR is still mainly shaped by social sciences often leading to a subordination of other disciplines in research projects—contradicting the call for a balance of disciplines as condition for a successful interdisciplinary collaboration (Castán Broto et al. 2009).

Besides, the above mentioned quote also refers to the design of research and describing positive effects on the practicalities of DTR. Emphasis is placed on creative approaches to research and competitive advantages over colleagues, institutions, and research processes. The following quotation from a postdoc illustrates this (G3): *"I work with physicists and with mathematicians, with cultural scientists and external, yes, businesspeople. Also, people from foundations. And that’s so great, how we can work together creatively and what ideas we have that we really [wouldn’t have] come up with alone in my political science swamp, I'll say."* Here, not only the cooperation with researchers from other scientific disciplines is underlined, but also with non-scientific people such as “*businesspeople*” and “*people from foundations*”. The latter explicitly enriches the scientific debate and the production of knowledge. This also leads to a more complex and integrative way of research: *trans*disciplinarity.

Of course, both an integrative understanding of inter- and transdisciplinarity call for a different underlying structure. Institutional contexts are needed to enable scientists at all career levels to acquire the relevant competencies, provide financial and time-wise resources, and promote career paths in interdisciplinary (and transdisciplinary) contexts (Mäkinen, Evans and McFarland 2020; Woiwode and Froese 2020). In this regard, various participants underline positive experiences with interdisciplinary DTR in non-university settings, where appropriate *“free spaces”* (Professors, G1) are seen.

Although we are focusing on the qualitative interpretation of the interviews it seems noteworthy how the categories are distributed: Regarding the challenges of interdisciplinary research, slightly more *individual* challenges are mentioned while concerning the opportunities researchers mentioned almost twice as often *structural* opportunities than their perceived individual opportunities (for percentages see Table 3). This means that individual researchers perceive interdisciplinary DTR as a field where opportunities for the disciplines and research projects in general can be found. But researchers themselves are rather confronted with the challenges. It hints to the need for a systemic cooperation between different generations of researchers: while the younger are at the forefront of scientific advancement, the more experienced must carve out robust spaces for innovation, that mitigate personal risks and challenges to allow individual progress. This effect is supported by the finding that generally challenges of interdisciplinary research are addressed more often in the discussions than opportunities as also shown below.

#### Differences Between Career Levels

Overall, mentions of challenges and opportunities of interdisciplinary DTR are distributed quite evenly across all three status groups as seen in Table 3 in the online appendix. However, there are some particularities. Across all career levels, challenges are addressed three times as often as opportunities, in the group of professors even four times as often. For the latter, the focus is on structural challenges. Postdocs, on the contrary, emphasize challenges regarding their career planning and positioning in the academic landscape. This reflects the fact that there are only a few permanent positions in the German academic mid-level faculties for scientists with a doctorate. Moreover, professorships—as one of the few opportunities for permanent employment—are still largely defined through mono-disciplines. This increases the pressure to focus on career planning within disciplinary boundaries as an interdisciplinary profile could be obstructive. PhD students mostly addressed personal challenges of interdisciplinary collaboration. Particularly, they focus on the demands placed on individual researchers and the hurdle of finding a common language within interdisciplinary teams. These results may reflect their everyday challenges: PhD students in DTR are presumably most affected by the very pragmatic, operational questions of interdisciplinary research, since they are the ones carrying out research in project contexts, grad schools, etc. on a most practical level.

## General Discussion and Practical Implications

As overarching question of this paper, we wondered how interdisciplinarity could and should be mobilized to further advance the development of the field of DTR. While interdisciplinary research is considered necessary for multi-faceted problem-oriented research, our study shows: the concept is complex, and its application and evaluation are still under debate and development. This applies particularly to emerging research fields such as DTR.

### Complexity Leads to Uncertainty

In DTR the boundaries between the understanding of multidisciplinarity and interdisciplinarity in its narrow sense seem to be blurred resulting in different practical approaches to interdisciplinary research. This does neither do justice to the richness of the concept interdisciplinarity, nor does it fully tap into its potential for impactful knowledge co-production. Consequently, researchers’ clarity about the term—at least in the context of the research project they are engaged in, at best in the institution the project is embedded in—is needed to facilitate and advance interdisciplinary collaboration.

Moreover, the interviewees mentioned more challenges than opportunities when it comes to interdisciplinary DTR. This is an indicator of yet unstable dynamics and processes in an emerging field of research—which might be especially challenging for early career researchers. However, results also show that even facing various challenges, digital transformation researchers of all career levels are willing to learn, to face uncertainties, explore the unknown in the shape of another discipline, and to apply different measures to help others to conceive and practice interdisciplinary research. This underlines their readiness for personal and professional growth as well as their wish to grow the field of DTR. Also, the higher proneness of the interviewed early career researchers to an integrative understanding of interdisciplinarity may advance the development of DTR—on condition that they find conducive conditions and career paths that motivate them to remain in the field (Woiwode and Froese 2020).

The high number of challenges mentioned by all our participants might give the impression that current researchers in DTR are not yet cognitively and socially fully equipped—or not convinced—to support the stabilization and consolidation of the field. The latter is partly supported by some of the interviewed researchers’ doubts that DTR will establish itself as an independent research field—and if so, then only as a "*meaningless collective term for an extremely heterogeneous field*" (Professor, G2). Until now, some regard digital transformation as being researched as an "*integral part of all possible research fields*" (Professor, G1) and *“application contexts”* (PhD student, G4), since the *"digital [in the future] will simply be the normal"* (PhD student, G6).

The present study widens the scientific understanding about how researchers of different career levels perceive, learn, and practice interdisciplinarity in DTR. It further provides valuable indications of how interdisciplinary research in an emerging field can be profitably shaped for practice as we will unfold in the following.

### Facilitating Interdisciplinary Collaboration

Disciplinary expertise is essential for interdisciplinary collaboration if implemented in scientific contexts that are not dominated by structural, hierarchical foci only. Osborne (2013: 95) emphasizes: “Interdisciplinarity is what happens when disciplines collaborate and, hopefully, communicate, evolve, morph, synergise”. This links disciplinary expertise to interdisciplinary expertise instead of pitting them against each other as competing forces. It needs to be a continuous oscillation between the two. Following this thought, finding a mutual understanding of foreign disciplinary concepts, methods and perspectives was repeatedly mentioned by the interviewed researchers as central challenge.

These aspects point to a possible solution discussed earlier in proposing an added entity (‘facilitator’) responsible for building competencies, overcoming hierarchies, and fostering interdisciplinarity. This role may support the reduction of cognitive and structural challenges. In line with the reasoning of SIM it may open a room for safe experimentation with disciplinarity and interdisciplinarity as an “uprising from the periphery” (Jasanoff 2013: 100) leveraging the fact that from the margins of disciplinarity come ground-breaking questions (Foucault 1994).

Previous scholars have proposed criteria for maneuvering nascent fields of research and their corresponding complexities and inherent incertitude. Practical implications are being considered with specific roles to support the research endeavors: The concept of ‘boundary spanning’ (Tushman 1977; Bednarek et al. 2018), for example, refers to “the people and organizations who can move across communities and fields to enable effective knowledge exchange, including sharing language and values with diverse groups, and translating between them” (Christopherson et al. 2021: 51).‘Knowledge brokers’ (Bergenholtz 2011; Chan et al. 2017) are introduced to “facilitate the creation, sharing, and use of knowledge”, while they also “establish and maintain links between researchers and their audience” and “link know-how, know-why, and know-who” (Meyer 2010: 119). Both concepts carry great resemblance to our study participants’ call for a ‘facilitator’.

For this position we draw inspiration from the background of business innovation in alignment with the interviewees calls for elements of agile project management (Turner 2019) and Scrum (Lacey 2012) in DTR. Core ideas of such approaches revolve around working in iterative cycles in which the status, next steps, and goals are reflected and adjusted. Through these opportunities for exchange, collaboration and feedback, a common understanding is quickly established and translated into joint planning in an adaptive work process. The interviewees’ expressed needs for conducive organizational conditions could be met with this. Additionally, agile approaches include the early and regular involvement of various stakeholders—which points to the possibility that agile approaches meet the identified demand for participation from the outside to support inter- and transdisciplinary efforts of advancing the formation of a new scientific field.

The facilitator is specifically deployed in interdisciplinary settings to structure and guide the process of co-creation in yet uncharted environments. To further advancing the development of the field of DTR, the allocation of the collaborative responsibilities to a distinct role may liberate researcher to better apply their disciplinary expertise in interdisciplinary settings. The facilitator unburdens the people involved in the process from carrying the additional load of having to be familiar with all involved concepts and methods, while delivering content specific to their disciplinary expertise (Knapp Zeratsky and Kowitz 2016). This approach allows to build a rather integrated, evolving system that transcends limiting, conformist setups through an adaptive process instead of having to completely reject traditional structures (Laloux 2017). This serves the purpose of building trust in unfamiliar settings and mobilizing researchers to further unfold the key ideas of a SIM while working in emerging fields.

The aim of introducing a facilitator in DTR is to enable deep disciplinary expertise to unfold in interdisciplinary work settings, even if the ones involved are not yet completely accustomed to what the changed modes of knowledge co-production mean in practice. This could be tremendously helpful for tackling the challenges brought forth by our study participants: having to be fully fluent in the ‘language’ of interdisciplinarity, balancing co-productive work with individual career efforts, dealing with tensions between disciplinary constraints, hierarchy levels, reducing professional risks of early-career scholars, and maneuvering ambiguities in an emerging research field. This might further bypass the bottleneck of a researcher having to be “the type” for interdisciplinarity and encourage a fearless and productive error culture within the field opening it for innovation and thereby establishing additional relevance.

### Limitations

The study comes with several limitations. Most of the interviewed researchers stem from social sciences. Members of technical and natural disciplines are underrepresented. This may impact the diversity of perspectives on interdisciplinary research, its challenges, and opportunities in DTR. It also reflects the current development of the field. However, due to the sample size and the small disciplinary subgroups, these effects can neither be adequately documented, nor can differences between disciplines be identified.

Another limitation is that the group discussions were conducted online. Although, online formats can be used in pandemic times to comply to distance regulations and overcome geographically long distances between discussants they come with various challenges (e.g., Sander and Schulz 2015). Different studies found audio-visual group discussions to be less efficient in terms of quality and quantity of content as face-to-face discussions because more time is spent on socializing. The presence of moderators in face-to-face settings promises more control over the topic and possible deviations from the topic by dominant participants (e.g., Abrams et al. 2014). Also, possibilities for a lively and dense discussion are limited, as participants cannot interrupt each other due to the technical circumstances—a fact that certainly has advantages, too.

## Conclusion and Outlook

The demand for interdisciplinary collaboration in scientific research calls into question the self-image of disciplines and their interoperability. This especially holds true when keeping in mind that the massive increase in available data and the expanded possibilities of obtaining, combining, and evaluating them act as driving forces for further transformation processes. Those developments have the potential to sustainably change disciplinary boundaries while creating new areas of research—under the condition that also cognitive boundaries on the researchers’ personal level can be overcome (Woiwode and Froese 2020). However, the higher the degree of specialization and potential fragmentation in a field of research such as DTR, the more necessary is interdisciplinary—or sometimes even transdisciplinary—interaction. Without this interaction, at most, highly specialized problems can be solved, instead of the ones that are most compelling for science and society. Those kinds of problems are no longer doing us the favor of defining themselves in a disciplinary way (Mittelstrass 2012: 10). And even if interdisciplinary collaboration does not necessarily guarantee innovation and the discovery of new knowledge, it can provide fertile ground for creativity (Weingart 2000)—not only in DTR.

## Data Availability

The raw data that support the findings of this study are available on request from the corresponding authors. The data are not publicly available as they may contain information that could compromise the privacy of research participants. However, interview guidelines and codebook are openly available in a public repository.

## Footnotes

1 In Germany, where the present study has been conducted, the term “Digitalisierungsforschung” is used. Internationally, the discussion about digital transformation has spawned various terms trying to specify these developments. Additionally, key terms are not being used universally in different languages. While *digitization* refers to the process of converting information in digital format, *digitalization* describes more complex effects of digital communication and media infrastructures for societal processes and structures (Brennen and Kreiss 2016). *Digital transformation* is defined “as a process of shifting an organization from old approaches to new ways of working and thinking through the use of digital, social, mobile, and emerging technologies” (Gorenṧek and Kohont 2020: 102) and predominantly used in the business context (e.g., Verhoef et al. 2019). We broaden its scope to the society as whole and use the term referring to the collectivity of requirements, effects, potentials, and risks of the changes that accompany digitization.

2 While some authors use the terms trans- and interdisciplinarity synonymously (e.g., Apostel 1972; Weszkalnys and Barry 2013), others draw clear distinctions. According to Wickson et al. (2006), only *trans*disciplinarity really transcends disciplinary borders by (1) focusing on ‘real-world’ problems, (2) providing iterative, reflective research processes that are responsive to the questions, settings, and research groupings, and (3) including collaboration between researchers as well as external actors.

3 By using the term “postdoc” we refer to PhDs who do not (yet) have a position as professor.

5 Online-Appendix

6 Online-Appendix

7 Table 2 in the online appendix provides an overview about the distribution of relevant codes (in percent) among the different career levels.

8 See Table 1 for more information about the respective group.

9 An overview about the percentual distribution of codes related to this research question is provided in Table 3 in the online appendix.
